# The Effect of Structured Education Training Program on Oral Health Awareness Among School-Going Children of Central India: A Cross-Sectional Study

**DOI:** 10.7759/cureus.27161

**Published:** 2022-07-22

**Authors:** Shalini Sinha, Sweta G Pisulkar, Sharayu Nimonkar, Chinmayee Dahihandekar, Hetal Purohit, Vikram Belkhode

**Affiliations:** 1 Prosthodontics, Sharad Pawar Dental College and Hospital, Datta Meghe Institute of Medical Sciences, Wardha, IND

**Keywords:** public health program development, central india region, school going children, oral health awareness, oral health training

## Abstract

Background

In order to curb the ever-increasing load of diseases related to the oral cavity, there is a call for generating organized school-based oral health education and training programs. It is proposed that there will be an emphasis on the primary care of oral health of school-going children proven, which is often neglected. This will be beneficial for the early detection, intervention and thus prevention of further debilitating conditions of the pathologies pertaining to the oral cavity with the assistance of the structured program suggested in this article.

Aim

The aim of the study was the evaluation of oral health programs for oral health awareness and knowledge among school-going children in the Central India region.

Settings and design

This is a cross-sectional study with measurements before and after the implementation of the oral care program.

Materials and methods

This cross-sectional study, approved by the Institutional Ethical Committee, Datta Meghe Institute of Medical Sciences, Wardha, has been done according to the STROBE (Strengthening the Reporting of Observational studies in Epidemiology) checklist. A study based on certain questions was carried out amongst the school-going children of Central India, especially the Vidarbha region. A total of 250 school-going children were enrolled in the study. A survey based on a questionnaire was carried out among the study participants in the age group of 12-16 years of age, which consisted of questions pertaining to knowledge of oral health and hygiene maintenance. The program consisted of presentation slides, role-plays, and demonstrations for inculcating the knowledge.

Result

A total of 200 study participants responded to the questionnaire. Overall, the baseline mean score of knowledge with scale was 2.80 ± 1.73 SD, which after the intervention was observed to be 10.70 ± 0.54 SD, which showed an increase. There was an aptly 60.4% increase in test scores related to oral health after the specific oral health inculcation program based on questionnaire execution which was statistically significant (p<0.001).

Conclusion

A properly constructed school children-based oral health education and training program induces better results in the oral health-related comprehension of students.

## Introduction

World Health Organization (WHO) in 2020 said, "Oral health is essential to general health and quality of life. It is a state of being free from mouth and facial pain, oral and throat cancer, oral infection and sores, periodontal (gum) disease, tooth decay, tooth loss, and other diseases and disorders that limit an individual’s capacity in biting, chewing, smiling, speaking, and psychosocial wellbeing” [1]. WHO determines oral health as a necessary entity for systemic health. Degraded dental health is observed in individuals or in the whole population as well as all the health system levels. The major causes of degradation of oral health are caries and periodontal disease, which act as major factors leading to a decline in the general health of an individual [2].

In developing countries like India, there is finite access to services that are related to oral health, as ailments of teeth are often left without treatment or some teeth are extracted due to pain or discomfort. Public health research has that a number of professional, and community preventive measures and individuals are effective in preventing most oral diseases. The incidence of oral carcinoma in India standardized by age is 12.6 per 100,000 population [3-5]. People ranging between the age group of 65-74 years of age show a 19% incidence of edentulism [6-8].

The frequency of dental caries and the mean dental caries occurring have reduced in children of urbanized civilizations [9]. Changes like these frequently impute to amend living conditions and way of life, effectual use of services related to oral health, execution of school-based oral health care programs, ratification of regular self-aid practices, and use of fluoride dentifrice [10-12]. In order to curb the ever-increasing load of diseases related to the oral cavity, there is a call for generating organized school-based oral health education and training programs. Despite multiple interventions, the oral health status among preschool children remains poor. Consequently, it is imperative to investigate their tooth brushing behaviour and routine oral hygiene practices which are very important yet neglected behaviours [13-14].

The study had the purpose to evaluate the awareness regarding oral health and knowledge amongst the school-going children in the Central India (Vidarbha) region with the aid of a structured questionnaire. In addition to this objective, awareness assessment regarding oral health after the introduction of the structured school-built oral health education and training program is also a major purpose. It also aimed to measure the relation of apprehension of health related to oral cavity and knowledge and selected social and demographic variants. With the structured program suggested in this article, it is thus proposed that there will be an emphasis on the primary care of oral health of the school-going children proven to be beneficial for the early detection, intervention, and thus prevention of further debilitating conditions of the pathologies pertaining to the oral cavity.

## Materials and methods

Ethical Aspects Permission to execute this study was received from the Institutional Ethical Committee, Datta Meghe Institute of Medical Sciences, Deemed to be University (DMIMS (DU)/IEC/2018-19/7515). The study and the questions were clarified to the patients in a language they understood, and informed consent was obtained from the school's head before filling out the questionnaire. The research design was a cross-sectional study. This cross-sectional report has been made according to the Strengthening the Reporting of Observational studies in Epidemiology (STROBE) checklist (Table 1).

**Table 1:** STROBE Checklist. STROBE: Strengthening the Reporting of Observational studies in Epidemiology

	Item No	Recommendation
Title and abstract	1	The Effect of a Structured Education Training Program on Oral Health Awareness Among School-Going Children in Central India: A Cross-Sectional Study
		Abstract: Background: In order to curb the ever-increasing load of diseases related to the oral cavity, there is a call for generating organized school-based oral health education and training programs. It is proposed that there will be an emphasis on the primary care of oral health of the school-going children proven which is neglected to be beneficial for the early detection, intervention and thus prevention of further debilitating condition of the pathologies pertaining to the oral cavity with the structured program suggested in this article. Aim: Evaluation of oral health awareness and knowledge among school-going children in the Central India region. Settings and Design: Cross-sectional study Materials and Methods: This cross-sectional report has been made according to the STROBE checklist. A questionnaire-based study was carried out among school-going children of Central India. 250 school-going children were enrolled in the study. The questionnaire-based study was carried out among the study participants in the age group between 12-16 years of age which consisted of questions pertaining to knowledge of oral health and hygiene maintenance. Result: 200 study participants responded to the study. Overall, the baseline mean score of knowledge was 2.80 ± 1.73 SD which after the intervention was observed to be 10.70 ± 0.54 SD which showed an increase. There was an aptly 60.4% increase in knowledge related to oral health after the specific program execution which was statistically significant (p<0.001). Conclusion: A Properly constructed “School-based Oral Health Education” and training program obtains better results on the oral health-related comprehension of students was revealed from the present study.
Introduction		
Background/rationale	2	With the structured program based on the questionnaire for the assessment of the awareness regarding dental health suggested in this article, it is thus proposed that there will be an emphasis on the primary care of oral health of the school-going children proven to be beneficial for the early detection, intervention and thus prevention of further debilitating condition of the pathologies pertaining to the oral cavity.
Objectives	3	To evaluate the Oral Health awareness and knowledge amongst the school-going children in the Central India region using a structured questionnaire which formed the basis of the program for inculcating knowledge regarding oral hygiene. In addition, to compare and assess Oral Health apprehension, knowledge after the introduction of structured school build oral health education and training program. Also to measure the relation of apprehension of health related to oral cavity and knowledge and selected social and demographic variants.
Methods		
Study design	4	Cross-sectional study.
Setting	5	This cross-sectional report has been made according to the STROBE checklist. The questionnaire-based cross-sectional study was carried out among school-going children in the Central India region (Vidarbha). One school from each of the five academic regions of Central India was selected. A pilot research to determine the accuracy and efficacy of the questionnaire was estimated on 50 students before the viable study.
Participants	6	(a) : 250 school-going children in the Central India region who fulfilled the inclusion criteria were considered for the study. 17 schools were referred to as samples using a “stratified random sampling technique” stratified by location. Students from the age group of 12 to 16 years were randomly selected from each school. Inclusion criteria: The students who were willing to participate in the study. The students who are mentally and physically fit to participate in the study. The students with the ability to communicate well. Exclusion criteria: The students who were not willing to participate in the study. The students who are non-cognitive. Children who have a communication problem.
Variables	7	Intervention: 30 minutes communal lecture with the help of PowerPoint Presentation (PPTs) demonstrating ideal oral hygiene and teeth brushing techniques, Role-plays for 10 minutes regarding ideal patient-doctor interaction with respect to maintaining oral hygiene, and Video Demonstration and Demonstrations of oral hygiene measures for 20 minutes using Simulators for teeth brushing habits were conferred by the investigator. Measurable Outcome: The upsurge of this School-based Structured Oral Health Training was assessed with the help of alterations in the level of dental health-related awareness and information gained just after the fulfillment of the program. An upsurge in the program was determined by measuring the absolute learning gain (ALG) Score [13] and the percentage of alterations in the knowledge related to dental health which was measured on a score of 100. Data collection instrument A total of 15 knowledge-based questionnaires which were self-administered were designed in Marathi and English language. When the class period was in progress the questionnaire was circulated with due permission and guidance of the investigator and along with one of the school teachers. There were experts from the branch of public health dentistry, experts in social activities, and a group of deputy students from some of the school-going children who gauged the questionnaire for maintaining consistency of the same.
Data sources/ measurement	8	For each variable of interest, give sources of data and details of methods of assessment (measurement). Describe comparability of assessment methods if there is more than one group
Bias	9	Cross-examination was done by two individual observers to reduce the risk of bias.
Study size	10	With α=0.05, β=0.2, and power=0.8, the expected standard deviation (SD) is 2 and the mean difference is 3 which is obtained from the pilot. A total of 250 schoolchildren was the minimum sample size
Results		
Participants	11	(a) Response rate of 80% among 250 questionnaires was documented by 200 recorded responses. Amongst 200 students, 30.5% were in class 6, 32.7% students were in class 8 and 9 grade and 36.5% were in 10thgrade. The participants who were included in the study belonged to the age group of 12 to 16 years. The recorded mean age was 13.98 ± 1.094.
		(b) Give reasons for non-participation at each stage – absentee.
		(c) Consider the use of a flow diagram
Descriptive data	12	(a) Response rate of 80% among 250 questionnaires was documented by 200 recorded responses. Amongst 200 students, 30.5% were in class 6, 32.7% students were in class 8 and 9 grade and 36.5% were in 10^th ^grade.
		Outcome data	13	Report numbers of outcome events or summary measures.
Main results	14	(a) There were 11 knowledge-based questions. The question regarding the detrimental effects of soft drinks and indication to remove dental plaque and tartar, as well as dental anatomy, receive the lowest percentage. The knowledge related to teeth that were permanent which were included was analyzed as: The exact number of permanent teeth was known by 25% of the total participants. Only 30% of participants correctly knew about the protective layer on the teeth. The importance of routine dental checkups was known to 27% of participants. Brushing twice daily is imperative for proper maintenance of hygiene and caries prevention was known by about 50% of the population.
		Other analyses	15	Report other analyses done—eg analyses of subgroups and interactions, and sensitivity analyses - none
		Discussion		
Key results	16	A properly constructed “School-based Oral Health Education” and training program inflicts better results on the oral health-related comprehension of students was revealed by the study.
Limitations	17	It is a short-term study which contributes to its limitations. The study is only limited to the Central India region.
Generalisability	20	The benefit of the mentioned programs should further be expanded with the help of continuous oral health programs which are conducted in school and involve all the people who provide oral health, school personnel, students, and their parents.

The questionnaire-based cross-sectional study was carried out among school-going children in the Central India region. One school from each of the five academic regions of Central India was selected. Pilot research to determine the accuracy and efficacy of the questionnaire was estimated on 50 students before the viable study. Sample size calculation: With α=0.05, β=0.2, and power=0.8, the expected standard deviation (SD) is 2 and the mean difference is 3 which is obtained from the pilot. A total of 250 schoolchildren was the minimum sample size. Allocation concealment: 250 school-going children in the Central India region who fulfilled the inclusion criteria were considered for the study. Such schools were referred to as samples using the stratified random sampling technique. Students from the age group of 12 to 16 years were randomly selected from each school.

Inclusion criteria

The following were the inclusion criteria: students who were willing to participate in the study; students who are mentally and physically fit to participate in the study; and students with the ability to communicate well.

Exclusion criteria

The following were the exclusion criteria: students who were not willing to participate in the study; students who are non-cognitive; and children who have a communication problem.

Period of recruitment

The process of recruitment of participants for the study started in January 2018 after IEC approval.

Period of exposure

The recruitment process was completed after 4 months and then the exposure to various desired interventions started in May 2018.

Intervention

The following interventions were done: 30 minutes communal lecture with the help of presentation slides demonstrating ideal oral hygiene and teeth brushing techniques, role-plays for 10 minutes regarding ideal patient-doctor interaction with respect to maintaining oral hygiene, and video demonstration and demonstrations of oral hygiene measures for 20 minutes using simulators for teeth brushing habits.

The process of follow-up was started 3 months after the intervention. The process of data collection was started after 3 months of follow-up.

Measurable outcome

The upsurge of this School-based Structured Oral Health Training was assessed with the help of alterations in the level of dental health-related awareness and information gained just after the fulfilment of the program. The upsurge of the program was determined by measuring the absolute learning gain (ALG) score and the percentage of alterations in the knowledge related to dental health which was measured on a score of 100.

Data collection instrument

A total of 15 knowledge-based questionnaires were self-administered and designed in Marathi and English languages. When the class period was in progress the questionnaire was circulated with due permission and guidance of the investigator and along with one of the school teachers. There were experts from the branch of public health dentistry, experts in social activities, and a group of deputy students from some of the school-going children who gauged the questionnaire for maintaining consistency of the same.

There were two sections in the questionnaire: Section A: questions in this section were formulated to extract data on the social as well as the demographic factors of an individual which included questions regarding age, certain questions regarding nationality, grade, and educational level of the mother and father; Section B: oral health and hygiene routines along with tooth anatomy-based questions constituted 11 multiple-choice questions. The knowledge about the number of teeth which are permanent, the presence of protective layers on the tooth, the number of times one should brush their teeth per day, the ideal time period after which one should replace the toothbrush, and awareness about the main objective of the usage of dental floss and other dental aids, the awareness about routine dental check-ups, detrimental effects of soft aerated drinks in the oral cavity, dental caries, cause of tartar and plaque and gingival inflammation, which included their aetiology and signs and symptoms, and fluoride (anti-cariogenic element) present in toothpastes and its role in preventing caries.

## Results

A response rate of 80% among 250 questionnaires was documented by 200 recorded responses. Amongst 200 students, 30.5% were in class 6, 32.7% of students were in classes 8 and 9 and 36.5% were in class 10. The participants who were included in the study belonged to the age group of 12 to 16 years. The recorded mean age was 13.98 ± 1.094 years. There were 11 knowledge-based questions. The question regarding the detrimental effects of soft drinks and indication to remove dental plaque and tartar, as well as dental anatomy, received the lowest percentage. The knowledge related to teeth that were permanent was analysed as: the exact number of permanent teeth was known by 25% of total participants. Only 30% of participants correctly knew about the protective layer on the teeth. The importance of routine dental check-ups was known to 27% of participants. Brushing twice daily is imperative for proper maintenance of hygiene and caries prevention was known by about 50% of the population. The same percentage of students knew aptly the time period after which the toothbrush should be replaced. Variation of students’ response to oral health knowledge questions and correlation of mean score ± SD knowledge related to oral health after implementation questionnaire after the education program is given (Table 2)

**Table 2:** Comparison of pre- and post-test scores

Question (total number of schools =17)	Pre-Test (before implementation of program)		Post-Test (after implementation of program)		Χ2-value	p-value
	Yes	No	Yes	No		
Q1	55(27.5%)	145(72.5%)	200(100%)	0(0%)	227	0.0001,S
Q2	117(58.5%)	83(41.5%)	200(100%)	0(0%)	107.4	0.0001,S
Q3	68(34%)	132(66%)	200(100%)	0(0%)	197	0.0001,S
Q4	116(58%)	84(42%)	194(97%)	6(3%)	87.23	0.0001,S
Q5	46(23%)	154(77%)	200(100%)	0(0%)	250.4	0.0001,S
Q6	124(62%)	76(38%)	200(100%)	0(0%)	93.83	0.0001,S
Q7	26(13%)	174(87%)	194(97%)	6(3%)	285.1	0.0001,S
Q8	7(3.5%)	193(96.5%)	200(100%)	0(0%)	372.9	0.0001,S
Q9	0(0%)	200(100%)	200(100%)	0(0%)	-	-
Q10	0(0%)	200(100%)	185(92.5%)	15(7.5%)	344.2	0.0001,S
Q11	0(0%)	200(100%)	168(84%)	32(16%)	289.7	0.0001,S

**Figure 1:**
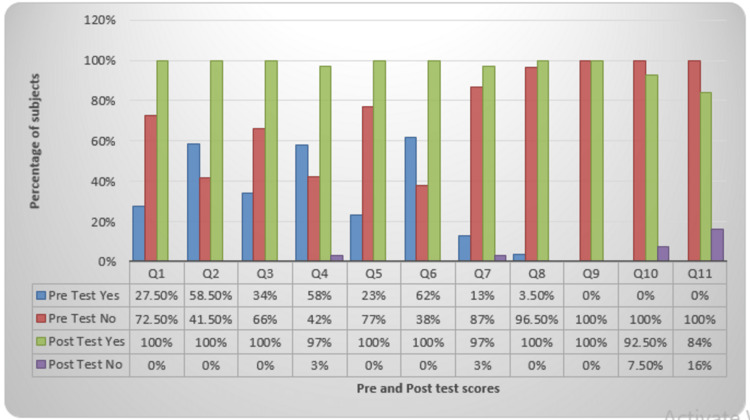
Comparison of pre- and post-test score

The implementation of the program included presentation slides, demonstrations, and role plays. Highly statistically significant improvements in the knowledge level related to oral health were seen after the implementation of the awareness program related to oral hygiene and health as evident from pre and post-test scores (p <0.001) (Figure 1).

## Discussion

This study was undertaken with the main objective of estimating of results of a school-based oral health education program and its implementation. The basis for this evaluation was oral health-related knowledge amongst children of school-going children in the Central India region which was carried out with the help of a structured questionnaire. The study results revealed that the school-based oral health education program had a constructive impact on the individual’s gain of information related to dental health. Overall, the baseline mean score of knowledge was 2.80 ± 1.73 SD, which after the intervention was observed to be 10.70 ± 0.54, which showed an increase. There was an aptly 60.4% increase in knowledge related to oral health after the specific program execution, which was statistically significant (p<0.001).

Dwivedi et al conducted a study in India, Rajasthan, and found significant statistical outcomes in the uplift in the knowledge of dental hygiene habits after the implementation of educational program (p<0.05) [13]. In a similar way, Biesbroke et al in their educational intervention study which was carried out in Brazil found an increase in awareness regarding oral health on the basis of their program concerned with education (P<0.001) [14]. D'Cruz et al in their study came to a conclusion that oral health programs can be advantageous for increasing oral hygiene knowledge and related practices along with improvements in the health of gingiva [15]. School-based oral health education (OHE) programs conducted in Brazil [16], Madagascar [17], and Indonesia showed similarly encouraging results.

One of the most essential parts of general health is oral health and should be integrated into the daily routine. Having good teeth is not the only prerequisite that defines good health, but it is also necessary for the overall systemic health of a human being. Oral health is a broad spectrum that includes not suffering from any kind of chronic pain related to the oral or facial region. Also, the individual should be free of cancers related to the oro-pharyngeal region, should not have oral and facial lesions, devoid of congenital defects of the oral cavity per se, and disorders that harm the oral, dental, and craniofacial tissues, collectively included in the envelope term craniofacial complex [18].

Health knowledge and awareness are of little value when the change in resources and opportunities do not exist. A radically different approach is now needed to reduce oral health inequalities and promote population oral health. Clinical preventive measures and behavioural approaches are not effective at tackling oral health inequalities. Instead, coordinated and integrated action is needed on the underlying social determinants of health, that is, upstream action to improve living, working, and social conditions [19].

The School Oral Health Program was implemented in the school-going children of the Central India region and showed a significant improvement. This may be linked to collaboration with the school's directors and principles, who were very cooperative, which in turn facilitated the process of learning and created an education-friendly environment. It is a short-term study, which is a limitation. The study is only limited to the Central India region. There were differences noticed, which might be due to the non-static relationship between the one who inculcates the knowledge of health and the varied groups of students. This happened even when the same information on oral health was given to all participants. This can lead to unwanted outcomes in the intervention [20-22].

The majority of the studies on the impact of school dental health education programs are conducted in the southern part of India whereas very few studies are conducted in the northern and western parts, and no study has been conducted in the north-eastern part of India. Therefore, it is the responsibility of the health sector to gather data regarding the conduction of school dental education programs in this part of the country [23]. Moreover, parents' support and involvement in the child’s oral health are important in influencing the oral health of the child. The majority of the surveys that are reported in the literature are targeted at school-going children due to easy accessibility, which is not possible in preschool children [24]. Therefore, there is a need to initiate more dental awareness programs for parents and their children at the preschool set up also to assess as well as to spread dental health awareness in Indian society.

## Conclusions

A properly constructed school-based oral health education and training program obtains better results in the oral health-related comprehension of students, as revealed by this study. On a general note, students and common people are unaware of the malfunctioning of oral health and always ignore the hygiene of the oral cavity and its maintenance. So this study will help them with awareness and play a crucial role to emphasize more time for maintaining personal oral hygiene. The benefit of the discussed program should further be expanded with the help of continuous oral health programs that are conducted in schools and involve all who provide oral health, school personnel, students, and their parents. In addition to the lecture technique, the use of innovation such as role plays, video demonstrations, and the use of simulation models for reinforcements, and hands-on training for oral hygiene measures will enhance the effect and will definitely serve as a motivational tool for the children.
